# The Erythrocyte Fatty Acid Profile in Multiple Sclerosis Is Linked to the Disease Course, Lipid Peroxidation, and Dietary Influence

**DOI:** 10.3390/nu17060974

**Published:** 2025-03-11

**Authors:** Ljiljana Stojkovic, Slavica Rankovic, Evica Dincic, Maja Boskovic, Ana Kolakovic, Mariana Seke, Marija Takić, Maja Zivkovic

**Affiliations:** 1 Laboratory for Radiobiology and Molecular Genetics, VINČA Institute of Nuclear Sciences—National Institute of the Republic of Serbia, University of Belgrade, 11000 Belgrade, Serbia; 2 Institute for Medical Research—National Institute of the Republic of Serbia, University of Belgrade, 11000 Belgrade, Serbia; 3 Clinic for Neurology, Military Medical Academy, 11000 Belgrade, Serbia; 4 Medical Faculty of the Military Medical Academy, University of Defence, 11000 Belgrade, Serbia

**Keywords:** fatty acid profile, erythrocyte, multiple sclerosis, disease course, clinical parameter, lipid peroxidation, 4-HNE, *n*-3 supplementation

## Abstract

**Background/Objectives:** Dietary lifestyle, particularly the intake of fatty acids (FAs), may be useful in alleviating the key pathogenic processes in multiple sclerosis (MS); however, the data are still scarce, particularly with regard to the course of disease. Therefore, the objectives of this study were to investigate the erythrocyte profile of FAs in patients with relapsing-remitting (RR)MS and progressive (P)MS, and to examine whether dietary supplementation with *n*-3 PUFAs could influence the FA profile, according to the course of disease. **Methods:** The FA profile was determined in erythrocytes by gas–liquid chromatography, in 153 patients with RRMS and 69 with PMS, whereas the group on dietary supplementation with *n*-3 PUFAs consisted of 36 RRMS and 17 PMS patients. Individual FAs were quantified as a percentage of the total identified FAs and analyzed in relation to the demographic and clinical parameters. **Results:** Compared to RRMS, the PMS patients had higher saturated (S)FAs, *n*-7 mono-unsaturated (MU)FAs, and *n*-3 polyunsaturated (PU)FAs, and lower *n*-6 PUFAs. In the group on omega-3 supplementation, the only difference in FA profile was higher MUFA 16:1*n*-7 (POA) in PMS than RRMS patients. In PMS patients, there was a positive correlation of disability (EDSS) with the total SFA levels, whereby 16:0 (PA) correlated positively with EDSS and MS severity (MSSS). Also, in PMS, the MSSS correlated negatively with the total and individual *n*-6, and positively with the total and individual *n*-3 PUFAs. In PMS patients on *n*-3 supplementation, there was a negative correlation between MSSS and total *n*-6/*n*-3 ratio, and a positive one between MSSS and 22:6*n*-3 (DHA). The observed decrease in levels of circulating lipid peroxidation product 4-HNE in PMS patients was not found in the *n*-3 PUFA supplementation group. **Conclusions:** The present findings suggest that the changes in the levels of FAs and their correlations are specific for the course of MS. Detected FA profile differences can be influenced by *n*-3 supplementation, primarily in regard to SFAs and PUFAs, supporting an option for the use of dietary supplements in managing the clinical course and progression of MS.

## 1. Introduction

Fatty acids and their metabolites are the established contributors of central nervous system (CNS) chronic inflammation, degeneration, and demyelination, which represent the hallmark pathogenic processes in multiple sclerosis (MS) [1,2]. The first findings indicating the importance of fatty acids in MS date back to the early 1950s, showing a link between MS and animal-derived saturated fat consumption [3]. Onwards, the demonstrated deficiencies of polyunsaturated fatty acids (PUFAs) in the plasma lipids among MS patients [4] brought the interest to PUFAs in the etiology and treatment of MS.

Due to their multiple roles, different classes of fatty acids are widely distributed in the CNS. The saturated fatty acids (SFAs) and mono-unsaturated fatty acids (MUFAs) are mostly synthesized de novo, while PUFAs have a low CNS synthesis rate and therefore need to be replenished by peripheral blood, through diffusion over the blood–brain barrier [5,6]. The long-chain SFAs were stated as inflammatory and neurotoxic, explaining the positive correlation obtained between the peripheral blood mononuclear cell (PBMC) membrane SFAs and the severity of neurological outcome in MS [7]. The pathogenesis of MS involves aberrant activation of the autoimmune Th1 and Th17 cells, with the suppression of regulatory T cells. Accordingly, the C14-C18 SFAs are able to suppress the differentiation potential of regulatory T lymphocytes and decrease the anti-inflammatory cytokine expression [8]. A decrease in SFA/PUFA ratio was associated with the maintenance of a non-pathogenic Th17 state, emphasizing that the fatty acid composition would decide the functional state of a particular T lymphocyte subtype [9]. Hence, an adequate intervention in fatty acid metabolism may have the immunomodulatory capacity, representing an additional option in the treatment of MS.

The *n*-6 PUFA-enriched Western diets were suggested to have an impact on high MS incidence in the Western world [10], whereas diets dominated by *n*-3 PUFAs, especially enriched in fish oils, were found to reduce the risk of developing MS and to improve the clinical outcome [11,12]. We previously demonstrated that the consumption of nutrients, such as *n*-3 and polyphenol-rich foods/beverages, could affect the composition of circulatory fatty acids, including PUFAs, in the healthy as well as the subjects at risk of chronic disease [13,14,15]. Erythrocyte membrane fatty acid composition and dietary fatty acid intake were demonstrated as predictors of inflammation in a general population [16]. In context of the CNS disorders, including MS, the general consensus is that *n*-3 PUFAs, such as 18:3*n*-3 (alpha-linolenic acid, ALA), 20:5*n*-3 (eicosapentaenoic acid, EPA), and 22:6*n*-3 (docosahexaenoic acid, DHA), have an anti-inflammatory role, given that dietary supplementation with *n*-3 PUFAs reduces the neuroinflammatory burden [17,18,19], which could be achieved by suppressing either the activation of the NLRP3 inflammasome [20] or the differentiation of Th17 cells [21]. In addition, the *n*-3-rich diet alleviated the score of experimental autoimmune encephalomyelitis (EAE) and reduced the CNS infiltration of IFN-gamma and IL-17-secreting CD4+T cells [22,23]. The anti-inflammatory effects of *n*-3 PUFAs may also be explained by the fact that they act as precursors of the anti-inflammatory specialized proresolving lipid mediators [24,25], able to inhibit the proinflammatory cytokine secretion from Th1 and Th17 cells [26]. Furthermore, the proposed neuroprotective role of *n*-3 PUFAs is founded on their ability to stimulate oligodendrocyte progenitor maturation, improve neuronal and oligodendrocyte survival, and attenuate demyelination, in experimental models [27,28,29,30]. It should be noted that feeding a salmon filet diet, rich in *n*-3 PUFAs, to cuprizone-induced demyelination models resulted in an alleviation of the MRI lesions and demyelination, while for a cod liver oil diet, also rich in omega-3 PUFAs, no such results were observed, highlighting the need for specification of the effective components [29].

In contrast to *n*-3, the *n*-6 PUFAs are considered to promote the inflammatory features of immune and glial cells, mainly because 20:4*n*-6 (arachidonic acid, AA) is a known precursor of proinflammatory lipid mediators, the eicosanoids, such as prostaglandins, thromboxanes, and leukotrienes [31,32]. Polarization of the immune cells towards a more pathogenic phenotype occurred after the ex vivo treatment of CD4+T cells with 18:2*n*-6 (linoleic acid, LA), which induced Th17 differentiation and proliferation, as well as the expression of their inflammatory cytokines [33]. The relation of *n*-6 PUFAs with neurodegeneration and neuronal dysfunction is supported by the discovery that 20:4*n*-6 (AA) could induce neuronal cell death and inhibit synaptic transmission in vitro [34,35]. In addition, the imbalance of cytokines could modulate myelin metabolism via *n*-6 PUFAs, given that the TNF-α signaling-mediated activation of phospholipase A2 and the following release of 20:4*n*-6 (AA) led to the activation of enzymes that cleave sphingomyelin [36].

Along with a high consumption of oxygen, the elevated content of easily peroxidizable fatty acids, primarily *n*-6 PUFAs, is one of the main reasons why the human brain is immensely susceptible to oxidative stress [37]. Therefore, the most toxic and one of the most abundant lipid peroxidation products is 4-hydroxynonenal (4-HNE) [38]. The excessive formation of 4-HNE-protein adducts causes a dysfunction of cellular proteins and is attributable to increased oxidative stress, neuroinflammation, demyelination, and neurodegeneration, the processes typical for both the pathogenesis and progression of MS [37,39,40].

Previous research showed that both circulatory and CNS tissue-resident fatty acids contributed to the pathogenesis of MS, linking metabolism with neuroinflammation, demyelination, and neurodegeneration. A considerable number of studies reported differences in the serum/plasma and erythrocyte profiles of long-chain fatty acids between MS patients and controls (reviewed in [41]). However, the changes in fatty acid levels and composition, as well as their possible therapeutic value, in relation to the clinically defined course of the disease are still insufficiently investigated. In a recent study, we analyzed the circulatory levels of lipid peroxidation products and showed that 4-HNE differed between the patients with relapsing-remitting (RR)MS and progressive (P)MS disease courses. Therefore, the aim of this study was to investigate the possible changes in the profile of long-chain fatty acids in the erythrocytes, according to the clinical course of disease—RRMS and PMS—and to test whether the fatty acid levels correlate with the levels of lipid peroxidation indicator, 4-HNE, and the clinical parameters. We considered erythrocytes as a preferable source, as these cells are able to provide the levels of fatty acids that are more stable with respect to dietary changes, hence varying more slowly than those in serum/plasma, and reflecting the average levels or metabolism of fatty acids over a longer time period [42,43]. We also aimed to examine whether dietary supplementation with *n*-3 PUFAs could influence the measured fatty acid levels and their correlations, in order to evaluate a possible use of the *n*-3 dietary supplements in managing the clinical course and progression of MS.

## 2. Materials and Methods

### 2.1. Study Subjects

The study cohort included 222 unrelated patients from Serbia: 153 with RRMS and 69 with PMS (54 secondary progressive (SP) and 15 primary progressive (PP)). Patients were recruited from the Clinic for Neurology of Military Medical Academy (MMA), Belgrade, Serbia, and the Neurology Clinic of University Clinical Center Nis, Nis, Serbia, during their regular visits to the clinics, 2022–2023. The disease was diagnosed to fulfill the revised McDonald criteria (2017) [44], and the course of disease was defined according to a clinical method [45], as RRMS, SPMS, or PPMS. The RRMS course was presented with relapses, as acute or subacute episodes of new or increasing neurologic dysfunction, followed by full or partial recovery—remission; SPMS included a disease course characterized by a progressive accumulation of neurological disability after the initial relapsing course, manifested as active or non-active (activity determined by clinical relapses, assessed at least annually, and/or MRI activity) and with or without progression (progression measured by clinical evaluation, assessed at least annually); PPMS comprised a progressive accumulation of disability from disease onset, manifested as active or non-active, with or without progression [45]. All details regarding the clinical data of the patients and the inclusion/exclusion criteria have been presented in our recent publication [46] and are included with Supplemental Table S1.

The patient group on dietary supplementation with *n*-3 PUFAs involved 53 patients, 36 RRMS and 17 PMS (14 SPMS and 3 PPMS). The same clinical criteria were applied for the recruitment of this group like for the initial study cohort. The *n*-3 supplementation comprised the daily use of 1000 mg of fish oil concentrate standardized to 30% *n*-3 fatty acids, 20:5*n*-3 (EPA) and 22:6*n*-3 (DHA) (DIETPHARM, PharmaS^®^, Belgrade, Serbia), during the period of 12 months.

### 2.2. Analysis of the Fatty Acid Profile of Erythrocytes

Fasting before a blood draw lasted for 12 h. The blood was collected into EDTA-containing tubes. Plasma and erythrocytes were separated by centrifugation at 3000× *g* for 10 min at +4 °C. The erythrocytes were washed three times in physiological solution and centrifugated at 3000× *g* for 5–10 min at +4 °C. After discarding the upper layer, the erythrocytes were washed and centrifugated once more at 3000× *g* for 10 min. The washed erythrocytes were kept frozen at −80 °C until further analyses.

The fatty acids from erythrocytes were isolated by direct methylation according to the method of Glaser [47]. Fatty acid methyl esters from erythrocytes (200 μL) were prepared by adding (2 mL) 3N HCl in methanol (0.05% BHT *w*/*v*) in a test tube for methylation. After vortexing, the mixture was placed in a drying oven for 1 h at 85 °C. After 60 min, when the test tubes have cooled to room temperature, 1.5 mL of n-hexane was added. In order to better separate the layers, the methylated samples were centrifuged at 3000 rpm for 10 min. The upper hexane layer was extracted and evaporated in a stream of nitrogen. Prepared fatty acid methyl esters (FAMEs) were dissolved in 20 μL of n-heptane and separated by gas chromatography in a Shimadzu chromatograph GC 2014 (Shimadzu Co. Ltd., Kyoto, Japan). The chromatograph was equipped with an injector and a flame ionization detector on an Rtx 2330 column (60 m × 0.25 mmID, film thickness of 0.2 μm, RESTEK, Bellefonte, PA, USA). The identification of FAMEs (from C:16 to C:22) was performed by comparing sample peak retention times with the certified calibration standard mixtures—PUFA-2 (Supelco, Bellefonte, PA, USA) and Supelco 37 FAME mix (Sigma chemical Co., St. Louis, MO, USA). Finally, individual FA is expressed as a percentage of the total detected fatty acids. The activities of D9D, D6D, and D5D were estimated based on the product/precursor fatty acid ratios (D9D 18:1*n*-9/18:0; D6D 20:3*n*-6/18:2*n*-6; and D5D 20:4*n*-6/20:3*n*-6). Also, the activities of elongase were estimated based on the product/precursor fatty acid ratios (elongase 18:0/16:0; elongase 18:1*n*-7/16:1*n*-7; elongase 22:4*n*-6/20:4*n*-6). The sum of 16:0 and 18:0 was defined as the total SFAs, while the sum of 16:1*n*-7, 18:1*n*-9, and 18:1*n*-7 was defined as the total MUFAs. The sum of 18:2*n*-6, 20:3*n*-6, 20:4*n*-6, and 22:4*n*-6 was used to define the total *n*-6 PUFAs, whereas the sum of 20:5*n*-3, 22:5*n*-3, and 22:6*n*-3 was used to define the total *n*-3 PUFAs. The *n*-3 index is expressed as a sum of 20:5*n*-3 and 22:6*n*-3 [48].

### 2.3. Statistical Analysis

The statistical analysis was conducted using the Statistica 8.0 software package (StatSoft, Inc., Tulsa, OK, USA). Differences in the distributions of categorical variables, according to the course of disease (RRMS and PMS patients), were attained by Fisher’s exact test. The normality of the distribution of continuous variables, encompassing the anthropometric, clinical, and molecular parameters, along with the assessed fatty acids, was tested using the Kolmogorov–Smirnov test with Lilliefors correction, and the Shapiro–Wilk test. Comparisons of the continuous variable values between the patient groups were performed using the *t*-test or Mann–Whitney U test, depending on whether the values had a normal distribution or not. For uniform presentation, the values of all the continuous variables are shown as the mean ± standard deviation. For estimation of the interactive effects of disease course (RR/progressive) and sex on the levels of the assessed fatty acids in MS patients, Factorial ANOVA was performed. Correlations of the clinical and molecular parameters with the fatty acid levels were estimated by the Pearson correlation test, whereby partial correlations were used when confounding variables (age, BMI) were included in the correlation analysis. In all tests, *p* < 0.05 values were considered significant.

## 3. Results

### 3.1. Study Population

The anthropometric, clinical, and molecular parameters of the patient cohort, according to the course of disease (RRMS and PMS), are presented in Supplemental Table S1. A description of the *n*-3 PUFA supplementation group, regarding the disease course, is given in Supplemental Table S2. The current study included comparisons of the following parameters between RRMS and PMS subjects: sex, age, BMI, onset age and duration of disease, EDSS, MSSS, and 4-HNE. In both the initial cohort and the group on *n*-3 supplementation, patients with PMS were older and had a longer disease duration, and, due to progressive disease, they also had higher EDSS and MSSS values (Supplemental Tables S1A and S2). Patients with PMS had decreased circulating 4-HNE (Supplemental Table S1B), while in the *n*-3 supplementation group, there was no difference in 4-HNE levels between RRMS and PMS patients (Supplemental Table S2). The *n*-3 supplementation did not affect the values of EDSS, MSSS, and 4-HNE, in either RRMS or PMS patients (*p* (Mann–Whitney U Test) > 0.05).

### 3.2. The Fatty Acid Profile in Patients with RRMS and PMS

The fatty acid profile of erythrocytes, with respect to the clinical course of MS, is shown in Table 1. In comparison to the RRMS group, the PMS group had higher total SFAs (*p* = 0.04) and *n*-7 MUFAs (16:1 POA (*p* = 2 × 10^−3^) and 18:1 cVA (*p* = 5 × 10^−4^)), and lower total *n*-6 PUFAs (*p* = 4 × 10^−3^) including the individual, 18:2*n*-6 LA (*p* = 0.04) and 22:4*n*-6 ADA (*p* = 0.03). The levels of 22:6*n*-3 DHA and total *n*-3 were increased in PMS (*p* = 0.03 and 0.04, respectively). Accordingly, the PMS patients had a lower *n*-6/*n*-3 ratio (*p* = 0.03) and higher *n*-3 index (*p* = 0.04) than the RRMS group did. In addition, they showed decreased activities of 18:1*n*-7/16:1*n*-7 elongase (*p* = 0.02) and 22:4*n*-6/20:4*n*-6 elongase (*p* = 0.04). The remaining comparisons resulted in no significant changes between the two investigated patient groups (Table 1).

The same analysis of the fatty acid profile was performed within the group of patients with RRMS and PMS who were consuming omega-3 supplements. The only differences obtained were an increase in 16:1*n*-7 POA levels (*p* = 4 × 10^−3^) and a consequential decline in 18:1*n*-7/16:1*n*-7 elongase activity (*p* = 0.01), in the PMS group compared to the RRMS group (Table 2).

### 3.3. Relationship Between the Fatty Acid Profile and the Anthropometric, Clinical, and Molecular Parameters, According to the Course of MS

The relationship between disease course, sex, and fatty acid profile was analyzed. There were no interactive effects between the disease course (RRMS/PMS) and sex with respect to the levels of either total or individual fatty acids (Factorial ANOVA/Multivariate Tests of Significance, *p* = 0.45, F = 1.00 or *p* = 0.49, F = 1.00, respectively).

We investigated correlations of the fatty acids with EDSS, MSSS, and 4-HNE, the parameters that have been denoted as different between the target groups of RRMS and PMS patients [46]. The tested correlations are shown in Table 3. Summing up, most of the differential correlations according to disease course were obtained between the clinical parameters and the SFAs, *n*-6 and *n*-3 PUFAs, in the patients with PMS (Table 3). In the patient group consuming *n*-3 supplements, there were only a few significant correlations, also observed in PMS patients, that linked MSSS with MUFAs and *n*-6 PUFAs (Supplemental Table S3).

The correlations adjusted for age and BMI are given in Table 4. After the adjustment, in patients with PMS, there were correlations of EDSS with the levels of total SFAs (r = 0.36, *p* = 0.005) and 16:0 PA (r = 0.42, *p* = 0.0009), while MSSS correlated negatively with the total *n*-6 PUFAs (r = −0.34, *p* = 0.009) and 22:4*n*-6 ADA (r = −0.48, *p* = 0.0001) and positively with the total *n*-3 PUFAs (r = 0.35, *p* = 0.007), 22:5*n*-3 DPA (r = 0.30, *p* = 0.02), and 22:6*n*-3 DHA (r = 0.32, *p* = 0.01) (Table 4a,b). In patients with RRMS, there were positive correlations of EDSS with 20:4*n*-6 AA (r = 0.19, *p* = 0.03) and 22:4*n*-6 ADA (r = 0.20, *p* = 0.03), as well as of 4-HNE with 16:0 PA (r = 0.20, *p* = 0.02) (Table 4b). In the group of patients on *n*-3 supplementation, the adjustment for age and BMI resulted in only two significant correlations, both in patients with PMS: a negative correlation between MSSS and total *n*-6/*n*-3 ratio (r = −0.56, *p* = 0.04), and a positive correlation between MSSS and 22:6*n*-3 DHA (r = 0.56, *p* = 0.048) (Supplemental Table S3).

## 4. Discussion

The fatty acid profiles of erythrocytes analyzed here revealed differences in the main classes of fatty acids, with respect to the clinical course of MS. Compared with RRMS patients, the PMS group had significant increases in SFAs, MUFAs, and *n*-3 PUFAs, and a decrease in *n*-6 PUFAs. These findings support the changes in profile of long-chain fatty acids specific to the clinical course of MS, in addition to previous studies that mainly investigated the MS onset [41]. A recent machine learning analysis of serum metabolomic data stratified 52 RRMS from 29 SPMS patients with high accuracy, showing higher MUFAs and lower PUFAs and *n*-6 levels in SPMS—results similar to ours [49].

We found that a rise in SFA and MUFA levels, that is, the total SFAs and individual *n*-7 MUFAs (16:1*n*-7 (POA) and 18:1*n*-7 (cVA)), was characteristic for the progressive course of MS. Several early studies obtained markedly higher levels of individual and/or total SFAs as well as 16:1 and 18:1 MUFAs, in red blood cells, but also the plasma/serum and cerebrospinal fluid of MS patients [4,50,51,52]. The higher SFAs could be linked to excessive neuroinflammation, since they are able to promote the proinflammatory signaling in macrophages and neutrophils [53], suppress the differentiation potential of regulatory T cells, and decrease the anti-inflammatory cytokine expression [8]. The neurotoxic nature of individual SFAs was proposed by the 16:0 (PA)-mediated reduction in the survival of neural stem/progenitor cells and hypothalamic neurons, and the negative impact on hippocampal neurogenesis [54]. Accordingly, in our study, 16:0 (PA) positively correlated with the severity of the neurological outcome in PMS patients, as measured by EDSS and MSSS. This correlation and the established increase in total SFAs, in terms of the stated proinflammatory and neurotoxic effects of SFAs, would be expected in the progressive course of MS. Yet, a previous study showed that higher PBMC membrane 16:0 (PA) reflected a better disease outcome, as demonstrated by the inverse correlation with the EDSS and functional system scores [7]. A similar inverse correlation was established between another SFA, 18:0 (SA), and MSSS in our PMS patient group, emphasizing the significance of various factors, such as disease status, type of sample for fatty acid analysis, fatty acid class and chain length, etc., in specifying the roles of individual fatty acids in MS. Still, an increase in total SFAs, together with the established positive correlation of EDSS with the total SFAs in PMS patients, indicates that this class of fatty acids contributed to the progression of MS.

Unlike the total SFAs, we proposed the impact of individual MUFAs, 16:1*n*-7 (POA) and 18:1*n*-7 (cVA), on the clinical course of MS, as we measured an increase in these *n*-7 fatty acids in PMS. An investigation into the fatty acid content of erythrocyte membrane phospholipids showed that 18:1*n*-9 and the total MUFAs had markedly higher concentrations in RRMS patients, compared to healthy controls [55], which, together with our results, suggest the differential effects of MUFAs with respect to the course and pathogenesis of MS.

The current study revealed a decline in total PUFAs in the PMS course, due to *n*-6 decline. All *n*-6 fatty acids were lower in PMS, compared to RRMS patients, with a significant lowering of the total *n*-6 PUFAs, 18:2*n*-6 (LA), and 22:4*n*-6 (ADA). In line with our findings, the erythrocyte, plasma, and cerebrospinal fluid fatty acid alterations were manifested as a deficit in the total PUFAs and 18:2*n*-6 (LA), and a less pronounced deficit in 20:4*n*-6 (AA), which was compensated with the increased levels of SFAs, in most studies on MS patients (reviewed in [41]). In addition, the metabolomics analyses of serum/plasma samples revealed that a decrease in 18:2*n*-6 (LA) levels could differentiate both SPMS and PPMS from the RRMS [49,56]. Yet, both studies, although comprehensive, had a limited sample size. A novel case–control study on a large sample (589 MS patients and 630 matched controls) also reported decreased total *n*-6 PUFA levels in patients [57]. There was an assumption that the low levels of PUFAs in patients’ erythrocytes, due to the impaired polyunsaturation process, could be compensated by an increase in SFAs, as an adaptive mechanism for maintaining normal fluidity of the cell membrane [4]. The decrease in PUFAs could also indicate an increased activity of phospholipase A2, resulting in a higher production of eicosanoids, thus depleting certain PUFAs from erythrocyte membrane pools [55,58]. Since it is known that the CNS takes up PUFAs from the peripheral blood because of the low synthesis rate [5], and that the changes in plasma/serum PUFA levels correlate well with the erythrocyte PUFA changes [41], it is possible that the decrease in erythrocyte *n*-6 PUFAs established here reflected their increased uptake by the CNS in PMS patients. This would result in enhanced CNS production of the predominantly proinflammatory *n*-6-derived lipid mediators [31,33], as well as an increased lipid peroxidation of *n*-6 PUFAs, known to be associated with inflammation, demyelination, and neurodegeneration [37,59,60].

Along with the total *n*-6 fatty acids, we found a reduction in 18:2*n*-6 (LA) in PMS, which represents one of the main sources of the reactive carbonyl species, 4-HNE, a known product of lipid peroxidation [38]. Hence, the currently detected decrease in 18:2*n*-6 (LA) could be linked with a decrease in circulating 4-HNE, observed in the same group of PMS patients and reported in our recent paper [46]. Namely, the presumed increased CNS uptake of *n*-6 such as 18:2*n*-6 (LA), in PMS patients could, under the conditions of enhanced oxidative processes, lead to an increase in 4-HNE and its consequent negative outcomes in the CNS cells, including oxidative stress and neurodegeneration, which are expected in the progressive course of MS [37,39,61]. In accordance with this possible explanation, the following correlations are obtained: the erythrocyte total *n*-6 fatty acids and 22:4*n*-6 (ADA) correlated negatively with both EDSS and MSSS in the PMS group, and the correlations with MSSS remained significant after adjustment for age and BMI, thus linking lower levels of circulating *n*-6 with a more severe clinical phenotype of the PMS course. In this context, a positive correlation of EDSS with 20:4*n*-6 (AA) and 22:4*n*-6 (ADA) in RRMS patients indicates that the increase in these *n*-6 fatty acids in circulation most likely would not be directly/causally related to an increase in EDSS. Similar to ours, previous studies showed that a reduction in serum 18:2*n*-6 (LA) and erythrocyte 20:4*n*-6 (AA) was more evident in MS patients with increased disease severity, according to the level of neurological disability and deterioration [62,63] or the activity of the disease [51].

The majority of previous studies compared MS patients with healthy individuals, noticing a decline in erythrocyte and plasma/serum *n*-3 fatty acid levels in MS patients (reviewed in [41]), but we detected higher total and individual *n*-3 PUFAs in erythrocytes of PMS, compared to RRMS patients. This finding may depict the specificity of the course of MS. In particular, an increase in total *n*-3 PUFAs, that is, 22:6*n*-3 (DHA), in the erythrocytes of PMS patients may reflect their reduced uptake from the circulation into the CNS, which could be specific for the progressive disease course. Consequently, the known beneficial effects of *n*-3, including the anti-inflammatory action [24,26], protective role in the survival of oligodendrocytes and neurons, and stimulation of remyelination [27,28,30], would be missed, which would also be expected in the course of PMS. In support of this assumption on reduced *n*-3 uptake from the circulation, we observed a positive correlation of erythrocyte total *n*-3, 22:5*n*-3 (DPA), and 22:6*n*-3 (DHA) with MSSS, exclusively in the PMS group. A study reported that serum 20:5*n*-3 (EPA) was associated with a more severe disease, estimated by an increase in disability in MS patients [64]. In line with the established changes in the levels of *n*-3 and *n*-6 PUFAs, we expectedly obtained a lower total *n*-6/*n*-3 ratio and a higher sum of 20:5*n*-3 (EPA) and 22:6*n*-3 (DHA) in PMS than RRMS patients.

In the correlation analysis, we found that, only in RRMS patients, 4-HNE positively correlated with 16:0 (PA) levels. It is known that 4-HNE is predominantly produced through the lipid peroxidation of PUFAs, and not SFAs, like 16:0 (PA) [38]. On the other hand, the stabilization of 4-HNE in a 1-palmitoyl-2-oleoyl-s*n*-glycero-3-phosphocholine (synthetic phosphatidylcholine containing 16:0 (PA)) bilayer was experimentally demonstrated, whereby 4-HNE had the ability to rapidly transfer to the extra- or intracellular space and was able to react with the cell membrane proteins and lipids as well as with proteins inside and outside the cell [65]. These facts support another role of 4-HNE, in cellular signaling pathways, which is generally known [38]. This possible role of 4-HNE, regarding the clinical course of disease and our current results, is suggested to be specific to RRMS, and requires further and more detailed research to be confirmed.

In addition, we investigated the RRMS and PMS patients who consumed the *n*-3 PUFA dietary supplements. The majority of differences captured in the group without supplementation were not detected, comprising the differences in PUFAs, both *n*-3 and *n*-6, and SFA levels as well, whereby the only significant change was an increase in 16:1*n*-7 (POA) levels in the PMS, compared to the RRMS. According to the course of disease, the subjects on *n*-3 supplementation showed no difference in 4-HNE levels, which we found to be in line with the lack of changes in PUFAs, especially the *n*-6, as declared to be prime sources of 4-HNE [38]. Similar to the results of the analysis of fatty acid levels, we confirmed that the majority of the revealed significant correlations were lacked in the *n*-3-consuming group, both in RRMS and PMS patients. We found a few correlations of the MUFAs, exclusively in the group on *n*-3 supplementation: MSSS correlated positively with the total MUFAs and 18:1*n*-7 (cVA) in the PMS, while EDSS correlated with 16:1*n*-7 (POA) in the RRMS; yet, the significance of each correlation was lost after the adjustment for age and BMI. The present findings suggest that the stated changes in the levels of fatty acids and their correlations are specific for the course of MS, but can also be influenced by *n*-3 supplementation, primarily in regard to SFAs and PUFAs. This gives an eventual opportunity for use of the *n*-3-based supplements in contributing to the management of disease course and progression. The consumption of *n*-3 fatty acids and fish oils has been associated with a lowering in the inflammatory markers, relapse rate, and disability progression in patients with MS [12]—effects that could be achieved through generating more *n*-3 downstream bioactive mediators, able to suppress the inflammation [25] and stimulate the remyelination [28]. Nevertheless, our results in the group on *n*-3 supplementation should be taken with caution particularly due to the small sample size.

Although there is a limitation regarding the difference in the proportion of patients with RRMS and PMS, this study enrolled a considerable total number of participants, in comparison to most previous studies, which mainly investigated the fatty acid profiles between MS patients and controls and in relatively small sample groups, lacking the associations according to the defined clinical course of disease, which we revealed here [7,55,63,66]. The advantage of using erythrocytes as a source of fatty acids lies in the fact that the biological variability in the fatty acid levels in erythrocytes is several times lower than that of plasma [67], thus reflecting a relatively long-term tissue fatty acid status and avoiding the concern that some true associations may be missed [68]. Another strength is that we established course-specific associations of the fatty acid levels with the levels of the molecular indicator of fatty acid peroxidation (4-HNE), as well as the values of relevant clinical parameters (EDSS and MSSS), thus linking the fatty acid changes with the oxidative stress, disability, and severity of the disease. Even though the *n*-3 PUFA supplementation did not directly affect the values of clinical parameters, we proposed that the stated course-specific changes in the levels of the fatty acids and their correlations could be influenced by *n*-3 PUFA supplementation. The latter observation supports an option for the use of *n*-3 supplements in managing the course of disease, possibly based on modifying the key MS pathogenic processes to some extent, by changing the levels and action of diverse fatty acid-derived metabolites, including the peroxidation products like 4-HNE. Still, despite the evidence on the beneficial effects in the experimental models, the clinical trials reported some conflicting outcomes regarding the impact of *n*-3 PUFAs on disability progression in MS patients [12,69]. Several factors that could cause the controversial outcomes may comprise differences in patient cohort characteristics, like the sample size, the source and dosing of fatty acids, trial duration, clinical endpoint measurements, etc., hence emphasizing the importance of adjusting for all these factors when evaluating the study results. 

## 5. Conclusions

The current study proposes that the erythrocyte profile of long-chain fatty acids could be specific for the course of MS, introducing the circulatory fatty acids as candidate molecular indicators for differentiating between the clinically defined RRMS and PMS. We established course-specific associations of the fatty acid levels with the levels of the molecular indicator of fatty acid peroxidation as well as the relevant clinical parameters, thus linking the fatty acid changes with the oxidative stress, disability, and severity of the disease. We also suggested that the stated course-specific changes in the levels of the fatty acids and their correlations could be influenced by *n*-3 PUFA supplementation, supporting an option for use of *n*-3 supplements in managing the course of disease. Further research should clarify the precise roles of endogenous and exogenous PUFAs, along with other long-chain fatty acids, in the pathogenesis and course of MS. The extended research should particularly focus on the effects of diverse classes of fatty acid-derived lipid mediators, by investigating these effects simultaneously in the CNS/cerebrospinal fluid and the periphery, and taking into account as many abovementioned factors to adjust for as possible, when estimating the main study outcomes.

## Figures and Tables

**Table 1:** Fatty acid profile of erythrocytes in RRMS (*n* = 153) and PMS (*n* = 69) patients.

	FA	Mean		SD		*p*
		RRMS	PMS	RRMS	PMS	
SFA	16:0	26.05	26.19	0.89	1.18	0.35 ^#^
	18:0	18.67	18.80	0.90	0.90	0.33 ^#^
	Total	44.73	44.99	0.84	0.90	0.04 ^#^
MUFA	16:1*n*-7	0.15	0.19	0.08	0.09	0.002 ^&^
	18:1*n*-9	12.49	12.44	0.96	1.02	0.73 ^#^
	18:1*n*-7	0.97	1.05	0.13	0.19	0.001 ^&^
	Total	13.62	13.69	0.98	1.14	0.64 ^#^
*n*-6 PUFA	18:2*n*-6	11.54	11.11	1.47	1.29	0.04 ^#^
	20:3*n*-6	1.48	1.51	0.37	0.42	0.92 ^&^
	20:4*n*-6	18.96	18.83	1.52	1.69	0.57 ^#^
	22:4*n*-6	3.96	3.71	0.78	0.79	0.03 ^#^
	Total	35.93	35.17	1.90	2.11	0.004 ^&^
n-3 PUFA	20:5*n*-3	0.24	0.24	0.23	0.15	0.65 ^&^
	22:5*n*-3	1.51	1.55	0.45	0.40	0.13 ^&^
	22:6*n*-3	3.96	4.37	1.28	1.40	0.03 ^&^
	Total	5.72	6.16	1.73	1.71	0.04 ^&^
	*n*-6/*n*-3 ratio	6.83	6.21	1.99	1.94	0.03 ^#^
	20:5*n*-3 + 22:6*n*-3	4.21	4.60	1.46	1.50	0.04 ^&^
	Total PUFAs	41.66	41.33	0.99	1.42	0.04 ^#^
Estimated elongase activities	Elongase 18:0/16:0	0.72	0.72	0.05	0.06	0.80 ^#^
	Elongase 18:1*n*-7/16:1*n*-7	7.56	6.78	3.99	3.76	0.02 ^&^
	Elongase 22:4*n*-6/20:4*n*-6	0.21	0.19	0.04	0.04	0.04 ^#^
Estimated desaturase activities	D9D 18:1*n*-9/18:0	0.67	0.66	0.07	0.07	0.46 ^#^
	D6D 20:3*n*-6/18:2*n*-6	0.13	0.14	0.03	0.04	0.18 ^&^
	D5D 20:4*n*-6/20:3*n*-6	13.60	13.37	3.47	3.75	0.66 ^#^

FA—fatty acid; SFA—saturated fatty acid; MUFA—monounsaturated fatty acid; PUFA—polyunsaturated fatty acid; 16:0—palmitic acid (PA); 18:0—stearic acid (SA); 16:1*n*-7—palmitoleic acid (POA); 18:1*n*-9—oleic acid (OA); 18:1*n*-7—cis-vaccenic acid (cVA); 18:2*n*-6—linoleic acid (LA); 20:3*n*-6—dihomo-gamma-linolenic acid (DGLA); 20:4*n*-6—arachidonic acid (AA); 22:4*n*-6—adrenic acid (ADA); 20:5*n*-3—eicosapentaenoic acid (EPA); 22:5*n*-3—docosapentaenoic acid (DPA); 22:6*n*-3—docosahexaenoic acid (DHA); D9D—delta-9 desaturase; D6D—delta-6 desaturase; D5D—delta-5 desaturase; RRMS—relapsing-remitting multiple sclerosis; PMS—progressive multiple sclerosis; SD—standard deviation; ^#^ *t*-test; ^&^ Mann–Whitney U test; *p*-values < 0.05 were considered statistically significant. Individual FA levels were calculated as a percentage of total identified FAs and are presented as the mean with standard deviation.

**Table 2:** Fatty acid profile of erythrocytes in RRMS (*n* = 36) and PMS (*n* = 17) patients, representing a group on supplementation with *n*-3 PUFAs.

	FA	Mean		SD		*p*
		RRMS	PMS	RRMS	PMS	
SFA	16:0	26.08	26.00	0.82	0.84	0.75 ^#^
	18:0	18.55	18.71	0.85	0.67	0.50 ^#^
	Total	44.62	44.71	0.73	0.71	0.71 ^#^
MUFA	16:1*n*-7	0.15	0.19	0.14	0.06	0.004 ^&^
	18:1*n*-9	12.54	12.57	0.89	1.05	0.92 ^#^
	18:1*n*-7	0.97	1.03	0.10	0.11	0.07 ^#^
	Total	13.67	13.79	0.93	1.11	0.69 ^#^
*n*-6 PUFA	18:2*n*-6	11.27	10.94	1.21	1.37	0.39 ^#^
	20:3*n*-6	1.44	1.43	0.33	0.39	0.63 ^&^
	20:4*n*-6	18.70	18.92	1.55	1.43	0.63 ^#^
	22:4*n*-6	3.67	3.54	0.84	0.77	0.58 ^#^
	Total	35.08	34.83	2.45	2.32	0.40 ^&^
*n*-3 PUFA	20:5*n*-3	0.32	0.28	0.32	0.18	0.99 ^&^
	22:5*n*-3	1.75	1.69	0.62	0.46	0.92 ^&^
	22:6*n*-3	4.55	4.69	1.58	1.51	0.51 ^&^
	Total	6.62	6.67	2.22	1.99	0.77 ^&^
	*n*-6/*n*-3 ratio	5.83	5.78	1.77	2.12	0.93 ^#^
	20:5*n*-3 + 22:6*n*-3	4.87	4.98	1.85	1.65	0.55 ^&^
	Total PUFAs	41.70	41.50	0.87	1.34	0.52 ^#^
Estimated elongase activities	Elongase 18:0/16:0	0.71	0.72	0.05	0.04	0.57 ^#^
	Elongase 18:1*n*-7/16:1*n*-7	8.28	6.01	3.86	2.06	0.01 ^&^
	Elongase 22:4*n*-6/20:4*n*-6	0.20	0.19	0.04	0.03	0.38 ^#^
Estimated desaturase activities	D9D 18:1*n*-9/18:0	0.68	0.67	0.07	0.06	0.78 ^#^
	D6D 20:3*n*-6/18:2*n*-6	0.13	0.13	0.03	0.04	0.95 ^&^
	D5D 20:4*n*-6/20:3*n*-6	13.74	13.97	3.70	3.23	0.30 ^&^

FA—fatty acid; SFA—saturated fatty acid; MUFA—monounsaturated fatty acid; PUFA—polyunsaturated fatty acid; 16:0—palmitic acid (PA); 18:0—stearic acid (SA); 16:1*n*-7—palmitoleic acid (POA); 18:1*n*-9—oleic acid (OA); 18:1*n*-7—cis-vaccenic acid (cVA); 18:2*n*-6—linoleic acid (LA); 20:3*n*-6—dihomo-gamma-linolenic acid (DGLA); 20:4*n*-6—arachidonic acid (AA); 22:4*n*-6—adrenic acid (ADA); 20:5*n*-3—eicosapentaenoic acid (EPA); 22:5*n*-3—docosapentaenoic acid (DPA); 22:6*n*-3—docosahexaenoic acid (DHA); D9D—delta-9 desaturase; D6D—delta-6 desaturase; D5D—delta-5 desaturase; RRMS—relapsing-remitting multiple sclerosis; PMS—progressive multiple sclerosis; SD—standard deviation; ^#^ *t*-test; ^&^ Mann–Whitney U test; *p*-values < 0.05 were considered statistically significant. Individual FA levels were calculated as a percentage of total identified FAs and are presented as the mean with standard deviation.

**Table 3:** Correlations of (a) total fatty acids and (b) individual fatty acids with the clinical parameters of MS and lipid peroxidation indicator in RRMS (*n* = 153) and PMS (*n* = 69) patients.

**(a)**								
**Fatty Acids**	**RRMS**				**PMS**			
	**EDSS**		**MSSS**		**EDSS**		**MSSS**	
	**r**		**r**		**r**		**r**	
	***p***		***p***		***p***		***p***	
Total SFAs	0.02 0.82		−0.14 0.10		0.33 0.008		−0.06 0.64	
Total MUFAs	−0.07 0.45		−0.07 0.43		0.13 0.32		0.17 0.18	
Total *n*-6 PUFAs	0.08 0.35		0.14 0.10		−0.29 0.02		−0.30 0.01	
Total *n*-3 PUFAs	−0.05 0.56		−0.04 0.66		0.09 0.50		0.33 0.008	
Total *n*-6/*n*-3 ratio	0.04 0.65		0.05 0.60		−0.11 0.38		−0.36 0.003	
**(b)**								
**Fatty Acid**	**RRMS**				**PMS**			
	**EDSS**	**MSSS**		**4-HNE**	**EDSS**	**MSSS**		**4-HNE**
	**r**	**r**		**r**	**r**	**r**		**r**
	***p***	***p***		***p***	***p***	***p***		***p***
16:0, PA	−0.07 0.43	−0.10 0.26		0.24 0.004	0.41 0.0007	0.25 0.05		0.07 0.55
18:0, SA	0.09 0.30	−0.03 0.72		−0.14 0.10	−0.20 0.12	−0.37 0.003		−0.17 0.18
16:1*n*-7, POA	0.11 0.20	0.06 0.52		0.02 0.81	0.09 0.49	0.12 0.35		−0.03 0.81
18:1*n*-9, OA	−0.11 0.22	−0.08 0.38		−0.07 0.40	0.09 0.47	0.16 0.20		−0.15 0.23
18:1*n*-7, cVA	0.19 0.03	−0.01 0.93		−0.01 0.89	0.19 0.13	0.05 0.68		0.07 0.57
18:2*n*-6, LA	−0.15 0.09	0.005 0.96		0.06 0.45	−0.004 0.97	0.004 0.98		0.04 0.73
20:3*n*-6, DGLA	−0.003 0.98	0.02 0.85		−0.09 0.29	0.002 0.99	0.14 0.25		−0.09 0.47
20:4*n*-6, AA	0.18 0.04	0.13 0.14		0.03 0.68	−0.23 0.07	−0.22 0.08		0.14 0.27
22:4*n*-6, ADA	0.14 0.11	0.09 0.33		−0.11 0.18	−0.30 0.02	−0.45 0.0002		0.13 0.31
20:5*n*-3, EPA	−0.02 0.83	0.01 0.89		0.05 0.55	−0.02 0.86	0.24 0.06		−0.04 0.78
22:5*n*-3, DPA	−0.09 0.33	−0.14 0.10		−0.07 0.41	0.14 0.27	0.27 0.03		−0.17 0.17
22:6*n*-3, DHA	−0.03 0.71	0.003 0.97		−0.04 0.61	0.05 0.68	0.30 0.02		−0.05 0.66

SFAs—saturated fatty acids; MUFAs—monounsaturated fatty acids; PUFAs– polyunsaturated fatty acids; PA—palmitic acid; SA—stearic acid; POA—palmitoleic acid; OA—oleic acid; cVA—cis-vaccenic acid; LA—linoleic acid; DGLA—dihomo-gamma-linolenic acid; AA—arachidonic acid; ADA—adrenic acid; EPA—eicosapentaenoic acid; DPA—docosapentaenoic acid; DHA—docosahexaenoic acid; RRMS—relapsing-remitting multiple sclerosis; PMS—progressive multiple sclerosis; EDSS—expanded disability status scale; MSSS—multiple sclerosis severity score; 4-HNE—4-hydroxynonenal; non-normally distributed data were log(2)-transformed; r—Pearson’s correlation coefficient; *p*-values < 0.05 were considered statistically significant.

**Table 4:** Correlations of (a) total fatty acids and (b) individual fatty acids with the clinical parameters of MS and lipid peroxidation indicator, after adjustment for age and BMI, in RRMS (*n* = 153) and PMS (*n* = 69) patients.

**(a)**								
**Fatty Acids**	**RRMS**				**PMS**			
	**EDSS**		**MSSS**		**EDSS**		**MSSS**	
	**r**		**r**		**r**		**r**	
	***p***		***p***		***p***		***p***	
Total SFAs	−0.06 0.51		−0.15 0.11		0.36 0.005		−0.01 0.91	
Total MUFAs	−0.09 0.32		−0.09 0.31		0.13 0.32		0.19 0.16	
Total *n*-6 PUFAs	0.12 0.20		0.15 0.10		−0.25 0.06		−0.34 0.009	
Total *n*-3 PUFAs	−0.04 0.69		−0.03 0.74		0.03 0.82		0.35 0.007	
Total *n*-6/*n*-3 ratio	0.03 0.71		0.04 0.68		−0.07 0.61		−0.38 0.003	
**(b)**								
**Fatty Acid**	**RRMS**				**PMS**			
	**EDSS**	**MSSS**		**4-HNE**	**EDSS**	**MSSS**		**4-HNE**
	**r**	**r**		**r**	**r**	**r**		**r**
	***p***	***p***		***p***	***p***	***p***		***p***
16:0, PA	−0.16 0.08	−0.14 0.13		0.20 0.02	0.42 0.0009	0.27 0.04		0.09 0.52
18:0, SA	0.10 0.26	0.005 0.95		−0.08 0.35	−0.20 0.14	−0.37 0.004		−0.13 0.34
16:1*n*-7, POA	0.10 0.30	0.05 0.56		−0.0004 0.99	0.12 0.35	0.14 0.29		−0.04 0.76
18:1*n*-9, OA	−0.13 0.16	−0.11 0.24		−0.06 0.46	0.09 0.48	0.17 0.19		−0.20 0.13
18:1*n*-7, cVA	0.17 0.06	0.02 0.79		−0.03 0.72	0.19 0.15	0.07 0.61		0.19 0.16
18:2*n*-6, LA	−0.15 0.10	−0.03 0.76		0.10 0.26	−0.05 0.73	0.005 0.97		0.08 0.57
20:3*n*-6, DGLA	0.004 0.97	0.002 0.98		−0.06 0.48	0.006 0.96	0.14 0.28		−0.19 0.14
20:4*n*-6, AA	0.19 0.03	0.17 0.06		0.04 0.61	−0.16 0.22	−0.25 0.05		0.11 0.41
22:4*n*-6, ADA	0.20 0.03	0.10 0.28		−0.07 0.42	−0.25 0.05	−0.48 0.0001		0.15 0.27
20:5*n*-3, EPA	−0.01 0.88	0.02 0.84		−0.02 0.82	−0.05 0.70	0.22 0.09		−0.11 0.43
22:5*n*-3, DPA	−0.07 0.45	−0.13 0.17		−0.12 0.17	0.11 0.43	0.30 0.02		−0.14 0.31
22:6*n*-3, DHA	−0.02 0.83	0.01 0.93		−0.10 0.28	−0.0002 0.99	0.32 0.01		−0.04 0.76

SFAs—saturated fatty acids; MUFAs—monounsaturated fatty acids; PUFAs– polyunsaturated fatty acids; PA—palmitic acid; SA—stearic acid; POA—palmitoleic acid; OA—oleic acid; cVA—cis-vaccenic acid; LA—linoleic acid; DGLA—dihomo-gamma-linolenic acid; AA—arachidonic acid; ADA—adrenic acid; EPA—eicosapentaenoic acid; DPA—docosapentaenoic acid; DHA—docosahexaenoic acid; RRMS—relapsing-remitting multiple sclerosis; PMS—progressive multiple sclerosis; EDSS—expanded disability status scale; MSSS—multiple sclerosis severity score; 4-HNE—4-hydroxynonenal; non-normally distributed data were log(2)-transformed; r—Pearson’s correlation coefficient; *p*-values < 0.05 were considered statistically significant.

## Data Availability

The original contributions presented in this study are included in the article/Supplementary Materials; further inquiries can be directed to the corresponding author.

## Supplementary Materials

The following supporting information can be downloaded at https://www.mdpi.com/article/10.3390/nu17060974/s1. Supplemental Table S1: (A) Anthropometric and clinical parameters of MS patients (n = 222) with regard to disease course, (B) molecular parameters in patients with MS (n = 222) according to disease course; Supplemental Table S2: Anthropometric, clinical, and molecular parameters in the MS patient group on *n*-3 PUFA dietary supplementation, regarding the disease course; Supplemental Table S3: Correlations of the fatty acids with the clinical parameters and lipid peroxidation product in MS patients on supplementation with *n*-3 PUFAs.
