# Supplementary material for: The Erythrocyte Fatty Acid Profile in Multiple Sclerosis Is Linked to the Disease Course, Lipid Peroxidation, and Dietary Influence
Source: Nutrients. 2025 Mar 11;17(6):974. doi: 10.3390/nu17060974 (PMC11944439; doi:10.3390/nu17060974)
Supplement: Supplementary file 1 [file nutrients-17-00974-s001.zip › Supplemental Table S1 (revised).pdf]

Supplemental Table S1:

A) Anthropometric and clinical parameters of MS patients (n = 222) with regard to disease course.

Anthropometric and Clinical Parameters	RRMS n = 153	PMS n = 69	*p*-Value
Sex (women/men, n)	79/74	42/27	0.25 $
Age (years)	43.0 ± 9.8	49.1 ± 7.9	< 0.001 #
Body mass index (BMI, kg/m^2)	24.40 ± 3.92	24.19 ± 4.12	0.67 &
Smoking status (yes/no/no data, n)	51/97/5 †	24/42/3 †	0.88 $
Disease onset age (years)	32.6 ± 9.2	34.2 ± 10.2	0.43 &
Disease duration (years)	10.4 ± 5.9	14.8 ± 8.2	< 0.001 &
EDSS	1.8 ± 1.1	5.6 ± 1.2	< 0.001 &
MSSS	2.32 ± 1.79	6.22 ± 1.83	< 0.001 &
Total number of relapses	2.00 (0.00–17.00)	6.00 (0.00–18.00)	< 0.001 &
Fatigue (yes/no/no data, n)	75/61/17 †	54/10/5 †	< 0.001 $
Therapy (yes/no, n)	139/14	50/19	< 0.001 $

RRMS−relapsing–remitting multiple sclerosis; PMS−progressive multiple sclerosis; n−number of patients; EDSS−Expanded Disability Status Scale; MSSS−Multiple Sclerosis Severity Score; $ Fisher exact test; # *t*-test; & Mann–Whitney U test; values of continuous parameters are presented as mean ±standard deviation except forthe total number of relapses (presented as median, minimum and maximum); *p*-value: comparison PMS vs. RRMS; *p*-values < 0.05 were considered statistically significant; † patients with no available data were not included in the analysis.

B) Molecular parameters in patients with MS (n = 222) according to disease course.

**Molecular Parameters**	**RRMS n= 153**	**PMS n= 69**	***p*-Value**
Malondialdehyde (MDA, ng/mL)	98.94 ± 40.71	103.77 ± 67.50	0.89
4-Hydroxynonenal (4-HNE, pg/mL)	1848.24 ± 1115.45	1712.27 ± 1289.24	0.03
Hexanoyl-lys adduct (HEL, nmol/L)	12.68 ± 3.93	13.17 ± 3.77	0.39
Glutathione peroxidase 4 (GPX4, pg/mL)	2503.18 ± 1606.99	2442.53 ± 1349.04	0.83
*Total glutathione (GSH + GSSG, µmol/L)	44.46 ± 6.48	42.03 ± 11.26	0.006
*Reduced glutathione (GSH, µmol/L)	15.77 ± 7.30	16.35 ± 8.13	0.91
*Oxidized glutathione (GSSG, µmol/L)	14.35 ± 2.47	12.84 ± 3.41	0.003
*Reduced/oxidized glutathione (GSH/GSSG)	1.18 ± 0.69	1.34 ± 0.69	0.17
Iron (Fe, µmol/L)	14.63 ± 5.34	14.60 ± 4.63	0.85
Transferrin (Tf, g/L)	2.53 ± 0.36	2.42 ± 0.43	0.07
Ferritin (Ft, ng/mL)	65.71 ± 61.58	75.39 ± 116.71	0.56

RRMS−relapsing–remitting multiple sclerosis; PMS−progressive multiple sclerosis; n−number of patients; values of continuous parameters are presented as means ± standard deviations; *p*-value (Mann–Whitney U test): comparison PMS vs. RRMS; *p*-values < 0.05 were considered statistically significant; *analysis performed in a subgroup of 100 patients, 63 RRMS and 37 PMS.

Description of the MS study cohort

In the study cohort of 222 subjects, MS was diagnosed to fulfill revised McDonald criteria [https://doi.org/10.1016/s1474-4422(17)30470-2], and the course of disease was defined according to a clinical method [https://doi.org/10.1097/00019052-200106000; https://doi.org/10.1016/j.jns.2010.07.023; https://doi.org/10.1093/brain/aww173; https://doi.org/10.1212/wnl.0000000000000560]. A detailed questionnaire was filled out for each patient, based on clinical records and an interview with a neurologist, to provide accurate data on anthropometric and clinical parameters, which were determined at the time of peripheral blood sample collection. The anthropometric parameters included age, sex, body mass index (BMI), and smoking status. Clinical parameters included disease onset age, disease duration, EDSS, MSSS, the total number of registered relapses, and previous and ongoing therapy. Fatigue was taken into account as a self-reported, subjective parameter. The EDSS score, representing a measure of neurological disability based on clinical assessments, was used for the evaluation of the clinical severity of the disease [https://doi.org/10.1212/wnl.33.11.1444]. The progression of disability was determined by MSSS, which corrects the EDSS for disease duration [https://doi.org/10.1212/01.wnl.0000156155.19270.f8]. The main inclusion criteria were the following: diagnosed MS, disease duration of at least one year, and age between 18 and 65 years. Exclusion criteria were the following: relapse during the period of at least 30 days prior to the study enrollment and consequently, a recent corticosteroid treatment, clinically isolated or radiologically isolated syndrome, comorbidities for neurological diseases other than MS and autoimmune diseases, diagnosed malignancies, pregnancy and inability to sign informed consent. Patients having acute infections were also not enrolled in the study. All patients had similar dietary habits regarding the intake of the main food groups. Besides vitamin D, there were no continuous supplementations at least three months before blood collection that could influence the measured parameters.
