# Supplementary material for: The Erythrocyte Fatty Acid Profile in Multiple Sclerosis Is Linked to the Disease Course, Lipid Peroxidation, and Dietary Influence
Source: Nutrients. 2025 Mar 11;17(6):974. doi: 10.3390/nu17060974 (PMC11944439; doi:10.3390/nu17060974)
Supplement: Supplementary file 1 [file nutrients-17-00974-s001.zip › Supplemental Table S2 (revised).pdf]

Supplemental Table S2. Anthropometric, clinical, and molecular parameters in the MS patient group on n-3 PUFA dietary supplementation, regarding the disease course.

**Parameter**	**RRMS n = 36**	**PMS n = 17**	**p**
**Sex (women/men, n)**	17/19	13/4	0.07 $^{\$}$
**Age (years)**	39.97 ± 9.34	46.06 ± 5.69	**0.02** $^{\#}$
**Body mass index (BMI, kg/m^2)**	23.22 ± 3.45	24.48 ± 5.37	0.31 $^{\#}$
**Disease onset age (years)**	30.82 ± 9.01	30.47 ± 7.71	0.89 $^{\#}$
**Disease duration (years)**	9.35 ± 6.88	15.59 ± 7.78	**0.008** $^{\&}$
**EDSS**	1.74 ± 1.04	5.79 ± 1.10	**<0.001** $^{\&}$
**MSSS**	2.61 ± 2.05	6.56 ± 1.81	**<0.001** $^{\&}$
**4-Hydroxynonenal (4-HNE, pg/ml)**	1907.46 ± 1500.75	1551.83 ± 972.53	0.12 $^{\&}$

RRMS–relapsing–remitting multiple sclerosis; PMS–progressive multiple sclerosis; n–number of patients; EDSS–Expanded Disability Status Scale; MSSS–Multiple Sclerosis Severity Score; values of continual parameters are presented as mean ± standard deviation; $^{\$}$Fisher exact test; $^{\#}$T-test; $^{\&}$Mann-Whitney U test; p-values < 0.05 were considered statistically significant.
